# “When you are injected, the baby is protected:” Assessing the acceptability of a maternal Tdap vaccine based on mothers’ knowledge, attitudes, and beliefs of pertussis and vaccinations in Lusaka, Zambia

**DOI:** 10.1016/j.vaccine.2018.03.081

**Published:** 2018-05-17

**Authors:** Anna Larson Williams, Lois McCloskey, Magdalene Mwale, Lawrence Mwananyanda, Kenya Murray, Augusta R. Herman, Donald M. Thea, William B. MacLeod, Christopher J. Gill

**Affiliations:** a Boston University School of Public Health, Department of Global Health, United States; b Boston University School of Public Health, Department of Community Health Sciences, United States; c Right to Care, Zambia; d Zambian Center for Applied Health Research and Development, Zambia

**Keywords:** Pertussis, Maternal Tdap, Vaccine attitudes, Maternal and child health, Vaccine hesitancy, Vaccine acceptability, Zambia

## Abstract

**Introduction:**

Severe and fatal pertussis infections are concentrated among infants who are too young to be protected through routine vaccinations. Maternal Tdap (mTdap), which is now the standard of care in the US and UK, is considered to be the most effective way to address this gap in preventative care. Little is known about how pregnant women in low-resource settings might view mTdap. To inform strategies for mTdap implementation in these contexts, public health researchers sought to understand knowledge, attitudes, and beliefs toward pertussis and maternal vaccines and assess the barriers to vaccine acceptance.

**Methods:**

We conducted focus group discussions (FGDs) among mothers who participated in a longitudinal birth cohort study at the Chawama primary health center in Lusaka, Zambia. Since SAMIPS was not a clinical trial, but instead an observational cohort study, registration on clinicaltrials.gov was not required. Trained interviewers conducted the FGDs in January 2016 using a semi-structured interview guide, exploring participants’ knowledge, attitudes and beliefs toward pertussis and vaccines. We analyzed the transcripts using Nvivo v.11 software.

**Results:**

Fifty mothers participated across 7 FGDs. Mothers had limited knowledge of pertussis and vaccines, yet expressed generally positive views of vaccinating themselves and their children. Participants conveyed very little vaccine hesitancy around maternal vaccinations, and discussed how they could protect their children’s health. Mothers also highlighted barriers and facilitators to vaccine uptake, which included partner involvement, feelings of maternal authority over healthcare decision-making, and community rumors about Western medicine.

**Conclusion:**

Mothers viewed vaccinations as an important method to keep their children healthy, despite cultural myths and misconceptions about pertussis and vaccines. FGD results suggest that vaccine acceptability is high in Zambia, which is a critical factor to vaccine uptake. A strategy addressing myths and misconceptions should be prioritized if and when mTdap is introduced across low-resource settings.

## Highlights

• Qualitative data from Zambia demonstrates that maternal vaccine hesitancy is low.
• Limited knowledge of vaccines and pertussis did not reduce vaccine acceptability.
• Zambian mothers support a maternal Tdap vaccine to protect infant health.
• Community rumors and partner involvement impact mothers’ healthcare decisions.

## 1 Introduction

Whooping cough, caused primarily by *Bordetella pertussis*, is a highly contagious respiratory disease believed to pose a significant threat to child health worldwide [1], [2]. Based on data from the UK, a maternal Tdap vaccine (mTdap) has been shown to be highly effective at protecting infants between birth and 3 months of age if administered in the third trimester of pregnancy [3], [4], [5], [6], [7], [8], [9], [10]. In fact, a maternal vaccine strategy against pertussis is now recommended by UK Department of Health and the U.S. CDC [11], [12].

Pertussis is pervasive in Zambia and other low and middle-income countries (LMICs); in a recent study, we identified a cumulative incidence of 5.2 cases of pertussis per 1000 infants and 2.4 cases per 1000 person-months among infants in Lusaka Zambia [13]. One similar study in Pakistan found an incidence of 3.96 cases per 1000 infants, and another comparing HIV-infected and -uninfected mothers and their infants in South Africa revealed an incidence rate of 6.8 vs. 3.9 episodes per 1000 person-months, respectively [14], [15]. The high incidence of pertussis in several LMICs points to the potential value of an mTdap strategy. However, Zambia and other similar countries follow the WHO guidelines on maternal vaccinations, which currently recommend that the tetanus toxoid vaccine (TT) be the only vaccine administered during pregnancy [16], [17], [18]. While mTdap could supplant one or more doses of maternal TT, there has not been any attempt to canvas mothers about acceptability of such an intervention in Zambia.

To successfully implement an mTdap strategy in Zambia, health officials must first understand the knowledge, attitudes and beliefs around maternal immunizations, and be able to address any concerns or knowledge gaps in the community to effectively promote vaccine uptake. Some studies in higher income countries revealed strong levels of support for the maternal vaccine, but there have also been incidences of vaccine hesitancy, which have contributed to pertussis outbreaks [19], [20], [21], [22], [23], [24], [25], [26]. Support of maternal vaccines in LMICs has not been studied systematically. Our research aimed to assess the feasibility of implementing a maternal vaccination strategy against pertussis and potentially other pathogens in Zambia. Specifically, we addressed three main research questions: (1) what do mothers know about whooping cough, (2) what are mothers’ attitudes about vaccines in general and maternal vaccines in specific, and (3) what factors promote or block uptake of vaccines in their families and communities?

## 2 Methods

This qualitative analysis was embedded within a larger research project, the “Southern Africa Mother Infant Pertussis Study” (SAMIPS), which aimed to determine the incidence of severe and non-severe pertussis in Zambia by enrolling 1981 mother/infant pairs and following the dyad when the infant was 2 to 14 weeks old, collecting nasopharyngeal swabs at approximately three-week intervals and testing the samples for pertussis [13]. The SAMIPS cohort enrolled mothers aged 18–39 who did not use any immunosuppressive agents during pregnancy, and their otherwise healthy infants who were less than 2 weeks of age at the time of enrollment. SAMIPS was approved by the Boston University Institutional Review Board and the Zambian ERES Converge IRB. Since SAMIPS was not a clinical trial, but instead an observational cohort study, registration on clinicaltrials.gov was not required.

We conducted focus group discussions (FGDs) with mothers in Lusaka, Zambia in January 2016 at the Chawama Clinic, which serves a low-income population of approximately 150,000 individuals over an area of about 25 km^2^. A purposive sampling strategy was used to identify participants: Nurses chose women from the existing SAMIPS cohort whom they believed would be willing to participate. A researcher trained all FGD facilitators in qualitative methodologies and how to elicit responses from participants respectfully and in a way which reduces social desirability bias. Upon obtaining informed consent and explaining study procedures, the nurses facilitated the FGDs following a semi-structured guide. The interviews probed participants’ knowledge about whooping cough; vaccine attitudes and beliefs; and factors that they viewed as influential to vaccine uptake. Participants in the FGDs were not linked via personal identifiers. The sessions were recorded in Nyanja or in English, translated and transcribed into English, and then destroyed. Facilitators of the FGDs fluent in Nyanja and English validated the translation quality. Participants were compensated for their participation with refreshments and reimbursement for transportation costs.

A team of four researchers trained in qualitative analysis read the FGD transcripts and generated a consensus-based list of themes related to the research questions. Two of the four researchers coded each FGD using NVivo v.11 software to ensure that the transcripts were coded to systematically avoid errors.

## 3 Results

**Fig. 1:**
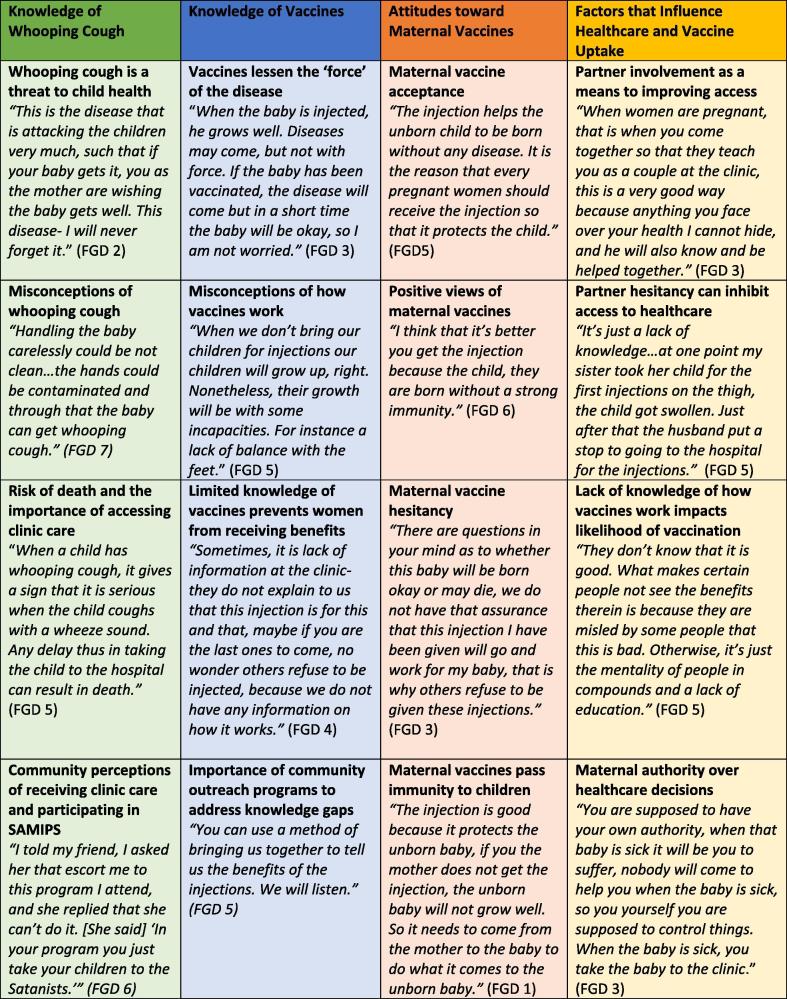
Vaccine attitudes with illustrative quotes, Chawama Clinic, January 2016.

We conducted 7 FGDs with a total of 50 participants. Each FGD had 6–8 participants and lasted between 1 and 1.5 hours. All of the respondents were women who lived in the Chawama compound, and the average age was 27. While 46% of participants reported completing some secondary education, only 10% completed secondary school. All women had received one or more doses of TT during the most recent pregnancy. Table 1 further describes the participants’ demographic characteristics. The participants echoed similar themes across FGDs, suggesting that the sample size was sufficient for saturation to be achieved. We present here the predominant themes we found to be salient to our research questions, detailed by exemplar quotes. Fig. 1 summarizes additional illustrative statements within each theme.

**Table 1:** Demographic characteristics of FGDs.

	Total N = 50
Age, mean (SD), years	27 (5.06)
Sex, No. (%)	
Female	50 (100%)
Race, No. (%)	
Black African	50 (100%)
Languages spoken, No. (%)	
English	1 (2%)
Nyanja	20 (40%)
Bemba	13 (26%)
Other	17 (34%)
Educational background, No. (%)	
None	2 (4%)
Some primary education	15 (30%)
Completed primary education	1 (2%)
Some secondary education	23 (46%)
Completed secondary education	5 (10%)
Some post-secondary education	0 (0%)
Completed post-secondary education	4 (8%)
Mother’s marital status, No. (%)	
Unmarried	4 (8%)
Married	46 (92%)
Living arrangements, No. (%)	
Father does not live with child	4 (8%)
Father lives with child	46 (92%)
Vaccine history, No. (%)	
Completed at least three doses of maternal tetanus toxoid	42 (84%)
Completed fewer than three doses of maternal tetanus toxoid	8 (16%)
Completed no doses of maternal tetanus toxoid	0 (0%)
HIV status, No. (%)	
HIV positive	17 (34%)
HIV negative	33 (66%)

### 3.1 Knowledge of whooping cough and perceived causes

Participant knowledge of pertussis was limited; many were able to list the disease symptoms, including cough and wheezing, but other associations with the disease were restricted to notions of the severity of the disease rather than insights into its causes. Some mothers knew that the disease is transmitted through the air, and can be passed from mother to infant. However, others thought that a contaminated air or environment could cause a child to contract the disease. One woman remarked, *“In the community we live in, the houses are too close and the toilets are too close to each other so when breathing, we breathe that air from the toilets and then you the mother gets sick and you give it to the baby”* (FDG 4).

The majority of mothers viewed whooping cough as a significant threat to child health, particularly noting the disease severity. One woman remarked, *“it is a bad cough and it can kill a child, when the child gets this cough you are supposed to bring in the baby so that they check him or her,”* (FGD 3). Despite limited knowledge of the causes of whooping cough, mothers demonstrated a strong understanding of the danger it posed to their children’s health.

### 3.2 Knowledge and perceptions of vaccines

The majority of mothers described vaccines as medicines designed to protect their children from dangerous diseases, and viewed them as supportive to overall health. In the words of one respondent, “*We bring children here to the clinic so that if the cough comes, you protect them in advance with medicine. Even if [disease] comes, it does not come with power because that child is already protected”* (FGD 2). And as one woman stated, *“Vaccines help our children to grow up well” (FGD 5).* When we probed further about how and why vaccines work to protect child health, respondents reported that they did not know or otherwise did not offer a response.

Some women understood vaccines to be general immune boosters that help to protect against any infection, regardless of which vaccine was administered. For example, one woman said that *“if you get that injection for tetanus even if you had malaria, you are protected because of that injection”* (FGD 4). Another asserted that vaccines are curative, rather than preventative, saying “*You go to the doctor and you are given medicine, you find that you get well, they give you an injection, you feel better, you find that when coming to the clinic you were not walking but after the injection you are able to walk”* (FGD 1).

### 3.3 Maternal vaccine attitudes

Women generally viewed the risk/benefit ratio of maternal vaccines similarly to those that are administered to infants. Discussing the benefits of maternal vaccines, one mother said she would have no problem getting vaccinated during pregnancy, *“because [vaccines] prevent children from being harmed by the diseases that can be transmitted just after birth. That is why we take them for injections to prevent some diseases so that they grow up well and healthy”* (FGD 6).

Only a small minority of women in the FGDs expressed concerns, which were mostly related to the vaccine not protecting the mother. One mother noted, *“I wouldn’t be okay with it if the vaccine that I have received only protects the child, because at a time that I am not feeling well, the child too will be affected*… *so a vaccine should protect both the child and me,”* (FGD 5).

### 3.4 Factors that influence vaccine uptake

Participants discussed the various factors that affect their decisions to vaccinate themselves and their children. We identified three most predominant factors below:

1. ***Male partner involvement in child healthcare.*** Participants noted that husbands expect to be informed of what happens at the health centers, even though it is almost always the mother who brings the child into the clinic for routine care. Some women argued that partners should be involved in the child’s healthcare to avoid problems at home. One woman remarked, *“if you never told him and he gets to know that this has been happening minus him knowing, he will ask why he was not told, and it brings problems to that home. So, a man is also supposed to know what happens at the clinic”* (FGD 2). Another noted, *“When it comes to bringing the children to the clinic, they have no idea at all, they do not know even what goes on here,”* (FGD 3). Many women expressed interest in getting their partners more involved in their child’s healthcare. One woman discussed partner involvement and support as a way to improve her access to healthcare: *“if a husband supports you, for example, when the baby is sick, we will go together the two of us we make a queue, one makes a queue for the book, the other one makes a queue somewhere else it becomes easier like that”* (FGD 4).
2. ***Maternal authority over healthcare decisions.*** Although women have voiced interest in partner involvement and have noted it as a factor that influences a woman’s decision to seek healthcare for her children, there were many women who expressed a personal authority over their children’s health. When asked whose opinions they valued most when making healthcare decisions like vaccinating their children, the majority of women said that they valued their own opinions over anyone else’s. Women also expressed a personal authority toward caring for their child, and saw it as a trait integral to motherhood. One woman said, *“If my husband refuses to let me come here it is up to me to decide because it is me who takes the baby, it is me who sees all the problems. He will go to work, I am supposed to bring my baby here so that he grows well,”* (FGD 3). Another woman added that it should be the woman’s responsibility to involve the partner as a way to wield her authority. She said, *“We the women should be the ones talking to our partner, so that he understands*…*we the women know that when our partner understands, he will remind you and he too will start coming here”* (FGD 5).
3. ***Community rumors and lack of knowledge.*** Many participants noted that a lack of understanding of why vaccines are necessary, both within the family and in the community at large, can impact willingness to receive vaccinations. One woman remarked, *“Maybe someone is sick of a cough, but you have no knowledge about whooping cough, what it is, how to treat this whooping cough so you will just stay at home, and will not go to the clinic, no wonder this cough has become very common in the community”* (FGD 4). Community rumors, such as an association between Western medicine (particularly blood draw procedures) and Satanism, exacerbate these knowledge gaps and may contribute to vaccine hesitancy in the community. Remarking on her husbands’ beliefs, one woman commented, *“he thinks that here, what we do, he thinks that those at the clinic are Satanic so they want to harm our children,”* (FGD 2). One mother noted that anecdotal fears in the community about adverse events from vaccines have prevented families from profiting from the beneficial aspects of vaccines. She said, *“Some people say that we should avoid our children getting used to injections or lest they become crippled*… *this scares some people making them miss the benefits of vaccines and the prevention therein,”* (FGD 5). Women noted that increased outreach between health professionals and the larger community helps to address these knowledge gaps. One woman commented, *“They do well to sensitize in the communities because we will not know, we will not know a lot of things unless when they come and tell us,”* (FGD 6).

## 4 Discussion

This research highlights Zambian mothers’ knowledge of, experiences with, and attitudes toward pertussis and vaccines. Although mothers had a limited knowledge of the causes of pertussis and how vaccines work, many highly valued maternal and neonatal vaccines. Mothers also reported commonly held beliefs about vaccines that conflict with allopathic medicine, some of which may stand in the way of vaccine uptake. For example, believing that vaccines are curative rather than preventative may not keep families from vaccinating their children, while assuming that vaccines act as general immunization boosters that cover all potential pathogens could either promote or discourage uptake.

Mothers conveyed an enthusiasm, rather than hesitancy, for maternal vaccines, suggesting that mTdap would be supported so long as healthcare professionals indicate the benefits to child health. There is a need to approach immunization in culturally sensitive ways to correct any misconceptions and alleviate concerns. Importantly, there is community support for educational initiatives such as Child Health Week, which has proven to be successful in Zambia not only in terms of vaccination rates, but also, as detailed in this research, in terms of acceptability [27].

Our findings indicate that the degree of partner involvement influences a woman’s ability to go to the clinic and vaccinate her child; a supportive partner facilitates access to healthcare, while a hesitant one could inhibit it. Partner involvement becomes more nuanced when considering that many women felt a personal authority over healthcare decision-making, expressing that it was their role to care for their child through clinic care, despite contrary familial opinions. Finally, mothers articulated concerns over community rumors, through which misinformation about pertussis and vaccines are spread, and which influence many women’s decisions to go to the clinic. These factors must be considered when implementing a new vaccine regimen, as they have the potential to facilitate or inhibit vaccine uptake.

It should be noted that our research held several limitations. First, due to the fact that the study population had underlying vulnerabilities and was already willing to participate in a study in a clinical setting, the participants’ understanding of vaccines and whooping cough may have been influenced by the consenting process, and be self-selected for more positive attitudes to vaccines. Second, this population already comes to the clinic regularly to receive care and 84% received at least three doses of maternal TT, so the full spectrum of opinions across the community may not be represented. However, we can argue that this could be consistent with country-level data; in a nation-wide household survey, 81.9% of mothers were found to have received TT during their last pregnancy, so therefore the opinions of this sample may not be dramatically different from the larger population [28]. Third, the purposive sampling strategy could limit the opinions represented, because study nurses selected women who would want to participate in a focus group. Finally, the data were all collected from one clinic in Zambia, and showing that these results are generalizable to other settings would require further research.

## 5 Conclusion

These limitations notwithstanding, it is our hope that data gleaned from this research can help Zambian public health officials to better address vaccinations at the population level. Our principal finding that mothers viewed maternal vaccines favorably suggests that little significant community-level vaccine hesitancy exists that would impede uptake of an mTdap vaccine. However, factors such as community myths and lack of partner involvement could counteract maternal acceptability. Other LMICs that rely on the WHO guidelines for vaccines may wish to consider the efficacy of the mTdap vaccine in the US and the UK, as well as the high levels of cultural acceptability found in this research. National immunization leaders should be aware of the importance of community sensitization as a useful tool to increase vaccine acceptability. Literature on health promotion efforts reveals that education and awareness initiatives are helpful to improving knowledge of the Tdap vaccine and reducing vaccine hesitancy [29], [30], [31], [32]. Applying this to the Zambian context, culturally-sensitive vaccination campaigns, already a standard in the Zambian health promotion tool kit, could encourage maternal immunization amongst Zambian women if mTdap vaccine were introduced.

Further research will be needed to confirm these findings and to assess their generalizability, and to continue to explore how misconceptions about vaccines, and anxieties about medical interventions from ‘Western medicine,’ could promote or discourage vaccine acceptance. Continuing to prioritize patient outreach and education will encourage more mothers to vaccinate their babies and themselves, which will play a role in reducing the burden of pertussis disease in this Zambian context and across other LMICs as they weigh the option of implementing an mTdap vaccine in their communities.

## Conflict of interest

The authors declared that there is no conflict of interest.

## References

[1] Yeung K.H.T., Duclos P., Nelson E.A.S., Hutubessy R.C.W. (2017). An update of the global burden of pertussis in children younger than 5 years: a modelling study. Lancet Infect Dis.

[2] Liu L, Oza S, Hogan D, et al. Global, regional, and national causes of child mortality in 2000–13, with projections to inform post-2015 priorities: an updated systematic analysis. Lancet (London, England). 2015;385(9966):430-40. 10.1016/S0140-6736(14)61698-6. 10.1016/S0140-6736(14)61698-6 25280870

[3] Amirthalingam G., Andrews N., Campbell H. (2014). Effectiveness of maternal pertussis vaccination in England: an observational study. Lancet..

[4] Hardy-Fairbanks A.J., Pan S.J., Decker M.D. (2013). Immune responses in infants whose mothers received Tdap vaccine during pregnancy. Pediatr Infect Dis J.

[5] Munoz F.M., Bond N.H., Maccato M. (2014). Safety and immunogenicity of tetanus diphtheria and acellular pertussis (Tdap) immunization during pregnancy in mothers and infants: a randomized clinical trial. JAMA.

[6] Vilajeliu A., Goncé A., López M. (2015). Combined tetanus-diphtheria and pertussis vaccine during pregnancy: transfer of maternal pertussis antibodies to the newborn. Vaccine.

[7] Dabrera G., Amirthalingam G., Andrews N. (2015). A case-control study to estimate the effectiveness of maternal pertussis vaccination in protecting newborn infants in England and Wales, 2012–2013. Clin Infect Dis.

[8] Terranella A., Asay G.R.B., Messonnier M.L. (2013). Pregnancy dose Tdap and postpartum cocooning to prevent infant pertussis: a decision analysis. Pediatrics.

[9] McIntyre P.B., Clark T.A., Mooi F. (2014). Pertussis vaccine in pregnancy—first dose for every infant?. Lancet.

[10] Mooi F., de Greeff S. (2007). The case for maternal vaccination against pertussis. Lancet Infect Dis.

[11] Pertussis: Summary of Vaccine Recommendations. Centers for Disease Control and Prevention. <http://www.cdc.gov/vaccines/vpd-vac/pertussis/recs-summary.htm>; Published 2016.

[12] Public Health England. Vaccination against Pertussis (Whooping Cough) for Pregnant Women-2016: Information for Healthcare Professionals; 2016. <https://www.gov.uk/government/uploads/system/uploads/attachment_data/file/529956/FV_JUNE_2016_PHE_pertussis_in_pregnancy_information_for_HP_pdf>.

[13] Gill CJ, Mwananyanda L, MacLeod W, et al. Incidence of severe and nonsevere pertussis among HIV-exposed and -unexposed Zambian infants through 14 weeks of age: results from the Southern Africa Mother Infant Pertussis Study (SAMIPS), a Longitudinal Birth Cohort Study. Clin Infect Dis 2016; Supplement. 10.1093/cid/ciw526 PMC5106616 27838668

[14] Omer S.B., Kazi A.M., Bednarczyk R.A. (2016). Epidemiology of pertussis among young pakistani infants: a community-based prospective surveillance study. Clin Infect Dis.

[15] Nunes M.C., Downs S., Jones S., van Niekerk N., Cutland C.L., Madhi S.A. (2016). *Bordetella pertussis* infection in South African HIV-infected and HIV-uninfected mother-infant dyads: a longitudinal cohort study. Clin Infect Dis.

[16] Organization W.H. (2017). Tetanus vaccines: WHO position paper. Wkly Epidemiol Rec.

[17] World Health Organization (2015). Pertussis vaccines: WHO position paper- August 2015. Wkly Epidemiol Rec.

[18] UNICEF USF for. MNT monitor: the campaign to save mothers and babies from tetanus. <http://www.who.int/immunization/diseases/08_fall_2005.pdf>.

[19] MacDougall D.M., Halperin B.A., Langley J.M. (2016). Knowledge, attitudes, beliefs, and behaviors of pregnant women approached to participate in a Tdap maternal immunization randomized, controlled trial. Hum Vaccin Immunother.

[20] Varan A.K., Esteves-Jaramillo A., Richardson V., Esparza-Aguilar M., Cervantes-Powell P., Omer S.B. (2014). Intention to accept Bordetella pertussis booster vaccine during pregnancy in Mexico City. Vaccine.

[21] Beel E.R., Rench M.A., Montesinos D.P., Mayes B., Healy C.M. (2013). Knowledge and attitudes of postpartum women toward immunization during pregnancy and the peripartum period. Hum Vaccin Immunother.

[22] Dempsey A.F., Brewer S.E., Sevick C., Pyrzanowski J., Mazzoni S., O’Leary S.T. (2016). Tdap vaccine attitudes and utilization among pregnant women from a high-risk population. Hum Vaccin Immunother.

[23] Chamberlain AT, Seib K, Ault KA, et al. Factors associated with intention to receive influenza and tetanus, diphtheria, and acellular pertussis (Tdap) vaccines during pregnancy: a focus on vaccine hesitancy and perceptions of disease severity and vaccine safety. PLoS Curr 2015;7. 10.1371/currents.outbreaks.d37b61bceebae5a7a06d40a301cfa819. 10.1371/currents.outbreaks.d37b61bceebae5a7a06d40a301cfa819 PMC4353696 25789203

[24] Ko H.S., Jo Y.S., Kim Y.H. (2015). Knowledge and acceptability about adult pertussis immunization in korean women of childbearing age. Yonsei Med J.

[25] Wiley K.E., Massey P.D., Cooper S.C., Wood N., Quinn H.E., Leask J. (2013). Pregnant women’s intention to take up a post-partum pertussis vaccine, and their willingness to take up the vaccine while pregnant: a cross sectional survey. Vaccine.

[26] (CDC) C for DC and P. Pertussis Epidemic—California; 2014. <https://www.cdc.gov/mmwr/preview/mmwrhtml/mm6348a2.htm> [accessed January 12, 2018].

[27] Fiedler J.L., Mubanga F., Siamusantu W., Musonda M., Kabwe K.F., Zulu C. (2014). Child Health Week in Zambia: costs, efficiency, coverage and a reassessment of need. Health Policy Plan.

[28] Central Statistical Office, Ministry of Health Zambia, ICF International. Zambia Demographic and Health Survey 2013–2014; 2014.

[29] Payakachat N., Hadden K.B., Ragland D. (2016). Promoting Tdap immunization in pregnancy: associations between maternal perceptions and vaccination rates. Vaccine.

[30] Kriss J.L., Frew P.M., Cortes M. (2017). Evaluation of two vaccine education interventions to improve pertussis vaccination among pregnant African American women: a randomized controlled trial. Vaccine.

[31] Goldstein S., MacDonald N.E., Guirguis S. (2015). Health communication and vaccine hesitancy. Vaccine.

[32] Chamberlain A.T., Seib K., Ault K.A. (2016). Impact of a multi-component antenatal vaccine promotion package on improving knowledge, attitudes and beliefs about influenza and Tdap vaccination during pregnancy. Hum Vaccin Immunother.

